# Changes in health, lifestyle, and wellbeing of children with type 1 diabetes and their parents during the pandemic

**DOI:** 10.1186/s40359-024-02102-z

**Published:** 2024-10-26

**Authors:** Afrah Alazmi, Simon Viktor, Mihela Erjavec

**Affiliations:** School of Human and Behavioural Science, Bangor University, Bangor, Gwynedd UK

**Keywords:** Covid-19, Chronic disease, Diabetes, Mental health, Wellbeing, Parenting

## Abstract

**Objective:**

The COVID-19 pandemic restrictions have substantially affected people’s health and rapidly changed their daily routines. This is a prospective study that investigated the impact of the pandemic on primary school children with Type 1 diabetes and their parents during the first lockdown in Kuwait.

**Methods:**

A questionnaire battery related to mental health, well-being, and lifestyle was administered at baseline in Summer 2019 (face-to-face, at a diabetes outpatient clinic) and at follow-up during lockdown in Summer 2020 (via telephone, in adherence with COVID-19 restrictions). Data were collected for 70 dyads with children aged 9–12 years.

**Results:**

Significant differences were found in most scores for both children and parents. Their mental health worsened to a higher level of depression, anxiety, stress, and a poor level of wellbeing. The average scores on the follow-up tests fell within a clinical range on these measures. Significant differences in their lifestyle, compared to before the lockdown, included decreased levels of physical activity and lower healthy core nutritional intake.

**Conclusions:**

Our findings indicate that the COVID-19 lockdown has had a significant psychological and possibly physiological impact on children with Type 1 diabetes and their parents. We conclude that there is a need for mental health support services focusing on these groups. Although full lockdown restrictions will have stopped in the past year, post-pandemic stressors may be expected to continue to adversely affect this cohort.

**Supplementary Information:**

The online version contains supplementary material available at 10.1186/s40359-024-02102-z.

Type 1 diabetes is a chronic disease that requires close medical attention and supervision of glucose monitoring [1]. Being diagnosed with Type 1 diabetes in childhood can lead to behavioural and mental health problems such as anxiety, depression, social anxiety, and lower self-esteem [2]. A diagnosis often leads to worry and stress-related responses regarding the complex care plan that needs to be adhered to by the patient and delivered by the parents [3]. A child with diabetes may potentially be anxious about how their condition will develop in the future, be fearful of leaving their house or communicating with others, and be prone to avoid social interactions with others [4].

A diagnosis may also affect the entire household in numerous ways financially, socially, or emotionally [5]. Coping with the disease can be challenging, especially for primary school aged children and their families [5]. Therefore, it is imperative that families learn to manage and cope with the effects that the disease might have on their children’s life-span development, yet there are few published studies regarding this situation, especially among younger, primary school aged children [6]. Such children may be more vulnerable to poorer mental health than their counterparts not diagnosed with a chronic illness [7]. In the existing literature, older children with Type 1 diabetes have been found to have poorer mental health such as anxiety, depression, aggressive behaviour, and attention problems than healthy children [8]. In a recent study, we have reported that 8–11 years old children with Type 1 diabetes had significantly higher scores in mental health problems, and lower scores in wellbeing, compared with a healthy control group [9].

Regarding their lifestyle habits, children with Type 1 diabetes suffer from poor sleep quality, and less physical activity due to hypocalcaemia phobia [10; 11]; those with higher Body Mass Index (BMI) show poorer mental health, including low academic self-esteem, depression, and anxiety [9]. Parents of children with Type 1 diabetes also suffer from anxiety and parental stress compared to parents of healthy children [12].

In March 2020, The World Health Organization [13; WHO] declared Covid-19 a pandemic. Also referred to as Coronavirus, it was a novel, highly contagious illness that has spread rapidly around the world [14]. The so-called lockdowns quickly changed people’s daily routines globally [15]. In Kuwait, the first lockdown period was from May to August 2020 [16]. There was reduced access to hospitals, and follow-up visits to outpatient departments were limited to emergency cases to help reduce the spread of the virus [13].

During the lockdown, measures were implemented that restricted individual freedoms, such as self-isolation and social distancing, and many people were forced to stay at home to reduce infection opportunities [17]. This was likely to have been detrimental to people’s well-being; for example, a Chinese study reported that the impact of the long period of separation from the world led to fear, guilt, and shame of being infected; these factors resulted in mental health issues, such as loneliness, panic, anxiety, depression, and sleep disorders [18]. Likewise, a Kuwaiti study of adults, administered via social media during the lockdown, showed that negative psychological impacts included elevated depression, distress, and poor quality of sleep [19].

Parents were burdened with additional caregiving roles during the pandemic; evidence suggests that parents respond negatively and more intensely to disasters compared to children, causing anxiety and posttraumatic stress [14]. Undefined periods of lockdown may have led to unprecedented impacts on parents’ mental health and well-being, with unknown effects on parent–child relationships [14]. It had been reported that patients with chronic illness had higher levels of depression, anxiety, and stress compared with healthy counterparts during the lockdown in Spain [20]. In Kuwait, a study conducted after pandemic lockdowns reported that health related quality of life declined in children and adolescents with Type 1 diabetes, and that their parents’ caring experiences were negative [21].

To our knowledge, there are no published prospective studies looking at the impact of the pandemic on the mental health, well-being, and lifestyle of children with chronic diseases such as Type 1 diabetes. The pandemic was rapid in onset and therefore, in most cases, the effects it may have had could only be estimated in retrospect. However, in summer of 2019, we collected data on mental health, well-being, and lifestyle from a large cohort of children with Type 1 diabetes and their healthy counterparts [see 9]. In summer of 2020, the first author was able to contact most of the parents in the diabetes group, all of whom consented to participating again.

The aim of the present study was to identify the impact of the COVID-19 pandemic on health, lifestyle, and well-being of children with Type 1 diabetes and their parents by re-examining the children’s and their parents responses to a questionnaire battery and physiological measures. The measurements reported in this study were administered before and during the Covid-19 lockdown, one year apart. We predicted that children with Type 1 diabetes would have higher levels of stress, anxiety, and depression during the pandemic compared to pre-lockdown. Regarding the parents, we also predicted that they would have higher levels of depression, anxiety, stress, fear, and shame due to the lockdown and isolation from the outside world. In the present paper, we report the changes in mental health, well-being, and lifestyle indices (e.g., eating habits and physical activity) for 70 primary age children and their parents.

## Methodology

### Design and sample

In July 2019, as part of a previous study looking at determinants of children’s mental health and well-being [9], we recruited 100 children (and their parents) from three Paediatric Diabetes Centres in Kuwait. Children and their parents were selected by a nurse and approached during their regular visits. The children were aged between 8 and 11 years and diagnosed with Type 1 diabetes; they had been undergoing intensive insulin treatment for at least 6 months via an insulin pump or multiple daily injections and did not have any other chronic disease. Follow-up measurement was conducted one year later, during the Covid-19 pandemic in July 2020, for 70 of the original dyads. We were unable to reach the remaining 30 dyads because their telephone numbers had changed, or they moved to another clinic. There were no systematic differences between the subgroup who could not be contacted and present sample on any of the baseline measures (all *p* > .05). Demographic characteristics of children and their parents (*N* = 70) are shown in Table 1 at follow up.

**Table 1:** Demographic characteristics of children and their parents (*N* = 70) at follow up

Therapy Type	59 insulin needles and 11 pumps
Children’s gender	35 girls and 35 boys
Median age	11 years (Range 9–12 years)
Median weight	35 kg (Range 27–82 kg)
Median height	139 cm (Range 125–163 cm)
Median BMI Percentile	76% (Range 20–99%)
Nationality	58 Kuwaiti and 12 non-Kuwaiti
Parents’ gender	64 mothers and 6 fathers
Parents’ median age range	35–44 years old
Median household size	6 members (Range 3–8 members)
Qualification	9 secondary school, 27 college, 24 bachelor’s degree, 1 master’s degree, 7 doctorate degree
Employment status	1 home carer, 15 unemployed, 1 self-employed, 1 employed part-time, 52 employed full-time

### Procedure

All study procedures were granted ethics approval by Bangor University research and governance committee (UK) and the Kuwait Ministry of Health.

At baseline, parents of children aged 8–11 years were approached by a nurse during their regular clinic visits; those interested in participating were taken to a quiet meeting room provided by the hospital for confidentiality and privacy. The researcher provided written and verbal information about the nature of the study and then parents signed the consent form. Parents and children were asked to complete a questionnaire battery containing measures related to their mental health, well-being, and lifestyle. The researcher assisted the children if needed. Self-report measures were completed in parallel by parents and their children. They were administered in the order listed in the Mesures section. Data collection took around half an hour, and participants could ask questions. At the end, they were debriefed and thanked. No incentives were offered for taking part.

A year later, in July 2020 during the Covid-19 lockdown, we obtained participants’ contact phone numbers from hospital records and invited them to participate in a follow-up. All parents who were contacted accepted this invitation; they were not offered any incentives for taking part. The researcher collected data via telephone calls with parents and children to avoid face-to-face contact and ensure compliance with lockdown restrictions and hospital regulations. The consent forms were sent via e-mail or smartphone application (WhatsApp). All participants completed the same questionnaire battery as in the baseline, in the same order. However, the experimenter read the questions to the participants, and parents were tested before their children. An additional brief set of questions regarding their lockdown experience was also administered. Data collection took longer than for baseline, partly because of the changes in the procedure necessitated by phone call, partly because the participants were keen to talk about their lockdown experiences. Many thanked the researcher for their interest.

### Measures

All measures were translated from English to Arabic either by their publishers, authors, or by the research team, following the appropriate guidelines [22]. They have been widely used in previous research and the Cronbach’s alphas for all measures at baseline and follow-up were above 0.40 (see Supplement file).

#### Physiological measures

HbA1c (Glycated hemoglobin) is the standard medical measure of average blood sugar concentration over the period of 8–12 weeks [23]. The International Society for Pediatric and Adolescent Diabetes (ISPAD, 2018) recommended values of less than 7.5 for children with diabetes; higher values would indicate poor diabetes management. HbA1c scores, height (in centimetres), and weight (in kilogrammes) were taken from the children’s hospital records at baseline and at follow-up.

#### Child self-completed measures

***Self-esteem.*** The Coppersmith Self-Esteem Inventory-School Form [CSEI; 24] is designed to measure attitudes toward the self on four subscales (general self, social self, home parent, and school academic). It’s a well-known instrument that’s been translated into many languages, including Arabic, and is widely used in clinical research and practice [24].

***Eating.*** The Kids Eating Disorder Survey [KEDS; 25] is a questionnaire that identifies eating disorders and attitudes through three subscales (body dissatisfaction, disordered eating, and binge eating). Test-retest data indicate that KEDS is reliable [25].

***Well-being.*** The WHO-5 Well-being Index [WHO-5; 26] measures health related quality of life in the last two weeks, with a higher score indicating better well-being. Parents also self-completed this questionnaire. WHO-5 is a short questionnaire with numerous uses. Aside from determining results in clinical studies, the WHO-5 is a useful instrument for screening for depression which has been translated into other languages, including Arabic [26].

***Mental health.*** The Revised Child Anxiety and Depression Scale [RCADS; 27] is a questionnaire that examines aspects of depression and anxiety in youth (subscales: social phobia, panic disorder, major depression, separation anxiety, generalized anxiety, and obsessive compulsive). Higher *T*-scores indicate more mental health related problems. DSM-IV refers to RCADS as user-friendly, publicly available, and translated into 16 languages other than English [27].

***Coping behaviour***. The Coping Questionnaire for Children and Adolescents [CODI; 28] has six subscales (acceptance; avoidance, cognitive palliative, distance, emotional reaction, and wishful thinking) with higher scores indicating coping-related problems. This questionnaire has been evaluated in six European countries and is intended to investigate the coping mechanisms used by children suffering from chronic illnesses like diabetes, asthma, or cystic fibrosis [28].

### Parent-completed child measures

***Mental health.*** The Child Behavior Checklist [CBCL; 29] consists of 29 items across four subscales (depressive problems; anxiety problems, anxious depressed, and withdrawn depressed), with higher raw scores indicating mental health problems. High values can be seen in the strength of the validity and reliability data across several languages and cultural contexts. Raw data is used for scoring each individual problem item [29]. Strengths and Difficulties Questionnaire measures emotional and behavioural difficulties [SDQ; 30]. The SDQ contains 25 items across six subscales (emotion symptoms; conduct problem, hyperactivity, peer problem, difficulty global score, and prosocial), with higher scores indicating mental health problems. The questionnaire has been translated into over 80 different languages. The questionnaire is particularly useful for researchers and clinicians who are concerned about service determinants and psychiatric events [30].

***Sleep quality.*** The Child’s Sleep Habits Questionnaire examined sleep behaviour [CSHQ-A; 31]; it contains 22 items across four subscales (bedtime, sleep behaviour, waking during the night, and morning wake up); a higher score means more disturbed sleep. The questionnaire has been translated into a variety of languages and used in numerous countries [31].

***Lifestyle.*** The Lifestyle Behaviour Checklist [LBCL; 32] uses 26 items that are focused on weight gain and eating activities. It consists of two subscales; behaviour associated with food (whining, arguing about, and refusing food), and physical activity and social situations, with higher scores on each subscale indicating a specific lifestyle-related problem. The questionnaire has strong internal consistency. The LBCL is beneficial for parents of obese children [32].

***Dietary behaviour.*** The Children’s Dietary Questionnaire measures food over the past seven days or the past 24 h [CDQ; 33]. It contains five subscales (fruit and vegetables, sweetened beverage, water, fat from dairy, and non-core food which means high fat, salt, or sugar food). Higher scores on subscales, except for fruit and vegetables and water, suggest an unhealthy dietary intake. This questionnaire was developed to be an easily administered and scoreable tool to monitor dietary intake and obesity [33].

***Physical activity.*** The Physical Activity Questionnaire for Children [C-PAQ, 34] estimates general levels of physical activity in children over a week (during free time, school time, and the weekend). Higher scores show higher levels of physical activity at each timepoint; higher sedentary behaviour scores are indicative of lower levels of physical activity. This questionnaire was chosen for the entire cohort due to the large age range of the participants and a lack of access to multimedia recall questionnaires at home [34].

### Parent-completed self-report measures

***Parental shame.*** The Other as a Shamer scale aims to measure external shame [OaS; 35]; a higher score means that parents may be feeling or experiencing more externally related shame. The shame scale has constantly been used throughout studies for its psychometric qualities and adoption to other populations [35].

***Parental coping behaviour.*** The Coping Health Inventory for Parents aims to appraise the behaviours that they are currently using to manage family life when they have a child with a chronic illness **[**CHIP; 36]. There are three subscales (Coping 1: cooperation and an optimistic definition of the situation, family integration; Coping 2: self-esteem and psychological stability, social support; and Coping 3: understanding the health care situation through communication with other parents and consultation with the health care-team). A higher score indicates more engagement with positive coping behaviours. Moreover, the Coping Health Inventory is used to predict how a family would adjust to a chronic stress condition; information regarding coping behaviors is required by the Resiliency Model of Family Stress, Adjustment, and Adaptation [36].

***Parental fear of hypoglycaemia.*** Hypoglycaemia Fear Survey [HFS-P; 37] contains 27 items across two subscales (behaviour and worry), with higher scores suggesting greater amounts of parental fear associated with managing their child’s possible hypoglycaemia. HFS-P is tailored to parents and caregivers of young children with Type 1 diabetes [37].

***Parental mental health.*** Depression Anxiety Stress Scales **[**DASS-21; 38] contains three subscales (depression; anxiety, and stress), with a total score over 14 suggesting a clinical condition may exist. Both exploratory and confirmatory factor analyses supported the factor structure of the DASS, demonstrating its satisfactory psychometric properties [38].

***Parenting behaviour.*** The Parenting Scale [39] contains three subscales (laxness; over reactivity, and verbosity) with higher scores indicating dysfunctional parenting, and low scores indicating good parenting. Five groups of researchers examined the parental behavior factor structure. Each study supported a two-factor solution (lax and overreactive) and demonstrated the validity of these factors across a range of constructs [39].

***Parental child feeding behaviour.*** The Child Feeding Questionnaire was used to assess parents’ feeding beliefs, practices, and attitudes related to child feeding [CFQ; 40]. It has seven subscales (responsibility; parental weight, chid weight, concern about child weight, pressure to eat, monitoring, and restriction), with higher scores indicating less adjustment in their intake in response to differences in caloric density of food. The CFQ is a tool for evaluating one aspect of the family environment: parents’ perceptions, beliefs, attitudes, and practices regarding child feeding, which are relevant to the development of obesity in children and have important clinical outcomes [40].

### Covid-19 impact measure

The parents’ questionnaire about Covid-19 impact in 2020 is a 12-item questionnaire that was designed specifically for this study to assess the impact of the pandemic on the children’s and parents’ daily life under lockdown. Answers were elicited on a five-point Likert scale (strongly agree; agree, neither agree nor disagree, disagree, and strongly disagree). Each item response was followed up with a further question about how Covid-19 had impacted the respondents in the relevant situations (individual items are listed in Table 2).

**Table 2:** Frequencies of the sample responses from parents regarding the impact of COVID-19 in 2020. *N* = 70

Item	Strongly agree	Agree	Neither agree nor disagree	Disagree	Strongly disagree
1- COVID-19 has had a negative impact on our family relationships.	4	53	3	10	-
2- COVID-19 has had a negative impact on our family finances.	-	23	3	44	-
3- COVID-19 has had a negative impact on me as a parent.	5	43	4	18	-
4- COVID-19 has had a negative impact on my mental health and well-being.	6	52	4	8	-
5- COVID-19 has impacted on how I manage my child’s diabetes.	4	53	5	8	-
6- COVID-19 has impacted on how my child manages their diabetes.	3	44	11	12	-
7- COVID-19 has had a negative impact on my child’s mental health and well-being.	7	53	3	7	-
8- COVID-19 has impacted on how medical services manage my child’s diabetes.	1	39	7	23	-
9- COVID-19 has had a negative impact on my daily routines (sleep, eating, exercise).	6	50	6	8	-
10- COVID-19 has had a negative impact on my child’s daily routines (sleep, eating, exercise).	3	55	3	9	-
11- COVID-19 events are making me worry about the future.	4	55	4	7	-
12- COVID-19 events have changed our lives in important ways.	5	53	3	9	-

### Data analysis and decision rules

Statistical analyses were performed using Statistical Package for the Social Sciences (SPSS) version 25. The distribution of scores for all scales and subscales were checked for skewness and kurtosis prior to undertaking inferential analysis; those that scored higher than ± 2 were investigated with non-parametric tests [41]. Thus, baseline vs. follow-up comparisons were performed by either repeated (paired or correlated) samples *t*-tests or Wilcoxon’s signed-ranks tests alongside the appropriate repeated measures effect size and power calculations. The raw data scores for The Child Behaviour Check List subscales were analysed instead of the *T-*scored data [42; 43]. The *T*-scored data for the RCADS were used.

Changes in parent and child scores from baseline to follow-up were represented by Reliable Change Indices (RCIs). According to Ferguson, Robinson and Splaine [44, p. 509], RCIs are a statistic that can be used to identify the magnitude of change score on a self-report measure that can be considered reliable. Hence, RCIs were used to identify significant changes on mental health variables from baseline to follow up in this study. The RCIs for the variables included in the regression analysis were calculated with the Leeds Reliable Change Indicator [45]. RCIs can be used in regression analysis to identity the strength and direction of the predictor variables [see 46].

All tests were two-tailed; even though we predicted that parents’ and children’s scores on mental health variables would increase during the pandemic, this was an unprecedented event and directional hypotheses could not be made based on the existing literature regarding specific measures. Cohen’s *d* statistics for repeated measures were used as indices of effect size [47].

### Data availability and additional information

Full anonymised data set is available online (10.5281/zenodo.7014722). Additional information is available in Supplement file that contains descriptive statistics for all scores; Cronbach’s alpha values; and clinical cut-offs for each measure used in the study, with counts of participants that fell into each category at baseline and follow-up.

## Results

### Changes in scores for children and parents

Across all measures, parents’ and children’s scores changed from the 2019 baseline to 2020 follow-up. Statistically significant changes are shown in the figures, and corresponding effect sizes are shown in Tables 3 and 4.

**Table 3:** *The Cohen’s d for the repeated measures t-tests*

	Effect Size (Cohen’s d)
SE Total Score	0.3*
SE General Self	0.4*
ED Items 1–7	0.1*
ED Body Dissatisfaction	0.5**
Well-being	1.2***
RCADS Social Phobia	0.7**
RCADS Panic Disorder	1.7***
RCADS Separation Anxiety	1.6***
RCADS General Anxiety	1.4***
RCADS Major Depress	1.6***
CBCL Depress Problem	1.6***
CBCL Anxious Depress	1.9***
CBCL Withdraw Depress	2.1***
CBCL Anxiety problem	2.2***
COP Accept	0.2*
COP Avoid	0.1*
COP Emotional Reaction	0.1*
COP Wishful Thinking	0.7**
Sleep Bedtime	0.6**
Sleep Behaviour	1.5***
Waking During the Night	0.8***
Morning Wake Up	1.0***
SDQ Emotion Symptoms	0.8***
SDQ Conduct Problem	1.6***
SDQ Hyper Activity	1.2***
SDQ Peer Problem	0.9***
SDQ Global Score	1.7***
SDQ Pro Social	0.4*
Shame Total	0.7**
COP Subscale1	0.3*
COP Subscale2	0.8***
COP Subscale3	1.5***
Parent Well-being	1.0***
HFS Behaviour	0.4*
HFS Worry	0.5**
DASS-21 Depression	2.0***
DASS-21 Stress	1.5***
DASS-21 Anxiety	1.6***
Parenting Style Laxness	0.5**
Parenting Style Over Reactivity	0.5**
Parenting Style Verbosity	0.3**
Parenting Style Sum	0.8***
Parental Feeding Perceived Responsibility	1.2***
Parental Feeding Perceived Parental Weight	0.1***
Parental Feeding Perceived Child Weight	0.3*
Parental Feeding Concern About Child Weight	0.8***
Parental Feeding Restriction	0.1*
Parental Feeding Monitoring	0.5**
Sedentary Behaviours Total Time in Minutes Weekday	0.8***
Sedentary Behaviours Total Time in Minutes Weekend	0.4*

**Table 4:** *The Cohen’s d for the wilcoxon signed Ranks tests*

	Effect Size (Cohen’s d)
HbA1C	0.3**
Lifestyle Food Score	0.2*
Lifestyle Physical Activity and Situation Score	0.3**
Fruits Eaten in The Last 7 Days	0.6*
Fruits Last frequency Week	0.3**
Veg Eaten in The Last 7 Days	0.5**
Veg Last frequency Week	0.6**
Non-Core Foods Last 7 Days	0.5*
Non-Core Foods Average Daily Portion	0.2*
Water Last 24 h	0.5**
Fruits Eaten Average Daily Portion	0.5**
Veg Eaten Average Daily Portion	0.5**
RCADS Obsessive Compulsive	1.1***

Small *; Medium **, and Large ***.

**Fig. 1:**
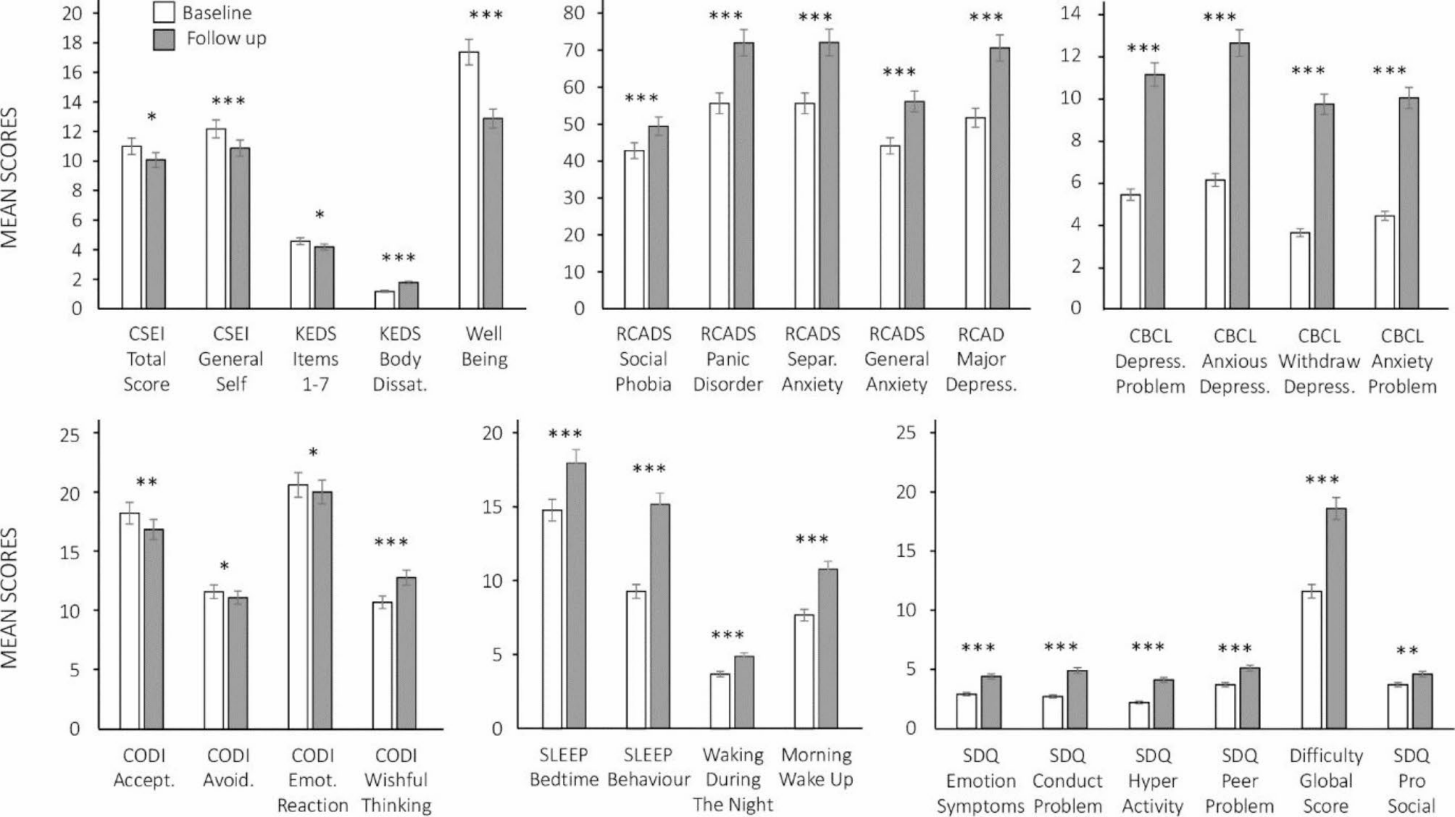
Child measures at Baseline (in white) and Follow-up (in grey). Bars represent means and standard errors. The graph at the top left shows CSEI total, CSEI general, KEDS 1–7 items, KEDS body satisfaction, and child well-being. The top middle graph shows RCADS subscales. The top right graph shows CBCL subscales. The graph at the bottom left shows CODI subscales. The bottom middle graph shows sleep habits subscales. The bottom righ graph shows SDQ subscales. All the results are significant with *p < .05, **p < .01, ***p < .001

**Fig. 2:**
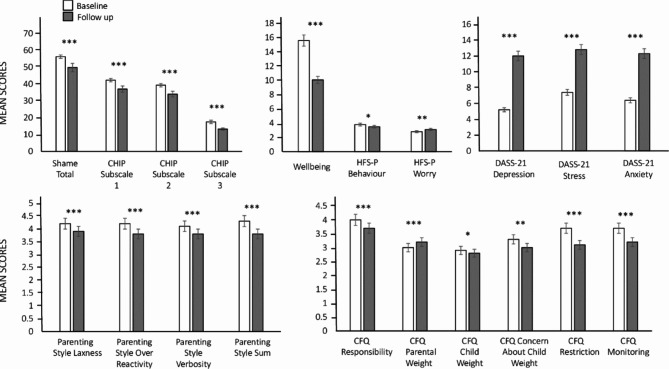
Parental measures at baseline (in white) and follow-up (in grey). Bars represent means and standard errors. The graph at the top left shows Shame total, and CHIP subscale. The top middle graph shows Well-being, and HFS-P subscale. The top right graph shows DASS-21 subscales. The graph at the bottom left shows Parenting subscales. The bottom righ graph shows CFQ subscales. All the results are significant with *p < .05, **p < .01, ***p < .001

**Fig. 3:**
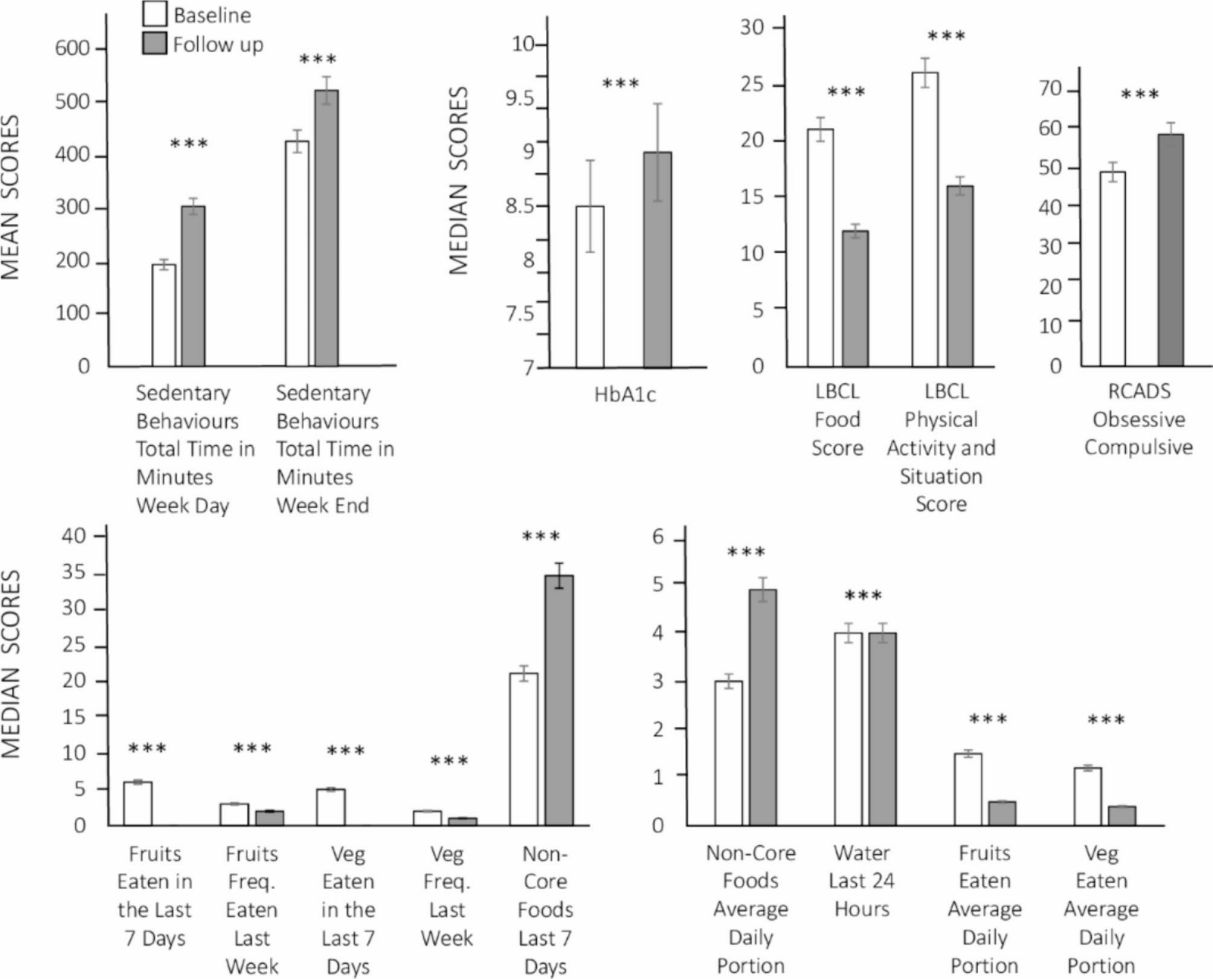
Lifestyle measures and HbA1c at baseline (in white) and follow-up (in grey). Bars represent mean or median scores and standard errors. The graph at the top left shows children’s physical activity subscales. The second graph shows HbA1c, and the third shows LBCL subscales. The top righ graph shows RCADS obsessive compulsive scores. The two graphs at bottom show the children’s dietary subscales. All the results are significant with *p < .05, **p < .01, ***p < .001

Figure 1 shows the mean scores for the child-completed self-report measures and parent-completed child measures at baseline and follow-up. Starting at the top left corner, it can be seen that children’s self-esteem (total and general), measured by CSEI, decreased. The same was also seen for children’s well-being scores, which shows that children had poorer self-esteem and well-being at follow-up compared to baseline. KEDS body dissatisfaction scores increased from baseline to follow-up, although children’s eating disorder scores (KEDS items 1 to 7) decreased. All child mental health scores (*T*-scored RCADS, raw CBCL, and SDQ subscales) showed increases with large effect sizes. These follow-up scores fell within a clinical range according to each measure’s cut-off point. Their CODI scores, pictured at the bottom left, indicate that children showed a decrease in acceptance, avoidance, and emotion reaction coping at follow-up; in addition, they also showed an increase in wishful thinking coping scores. The bottom middle graph shows the sleep habits subscales: there was an increase in sleep bedtime, sleep behaviour, waking during the night, and morning wake up scores, which all indicate that children experienced more sleep-related problems during the pandemic.

Figure 2 shows mean scores for the parental-completed self-report measures. The top left graph shows that shame total score decreased from baseline to follow-up. In a similar manner, CHIP scores for subscales 1, 2, and 3 also showed a decrease. There was a decrease in well-being scores from baseline to follow-up, with large effect sizes. The HSF-P scale scores showed a decrease, whilst the HFS-P worry scale scores showed an increase from baseline to follow-up. The top right graph shows the DASS-21 subscale scores for depression, anxiety, and stress. All three subscale scores increased from baseline to follow-up, with large effect sizes. These increases are also deemed problematic according to the cut-off scores provided by the scales’ authors (Lovibond & Lovibond, 1995). At the bottom left, the parenting subscale shows a decrease in score from baseline to follow-up. The bottom right graph shows the six CFQ subscales; scores on five out of six subscales show a decrease from baseline to follow-up, whilst the CFQ parental weight shows an increase.

Figure 3 shows mean scores for the children’s lifestyle measures and HbA1c values. The top left graph shows the children’s physical activity subscale scores. Sedentary behaviours are shown for weekdays and weekends; much of this time was spend playing video games and watching online content. Both scores show an increase from baseline to follow-up. Children’s activity levels were already low at baseline, and it is noteworthy that all parents reported that their children could not engage in any physical activity during the lockdown. The top middle graph shows that HbA1c values increased from baseline to follow-up, with about half of the sample scoring below 7.5 HbA1c in baseline, while only 12 children had such scores in the follow-up. This shows that many children’s diabetes status changed for the worse, from managed to unmanaged. The next graph shows the LBCL food score, and that physical activity and situation scores decreased from baseline to follow-up. RCADS score shows an increase in obsessive compulsive behaviour. The bottom graphs show children’s dietary behaviour – their intake of recommended foods and fluids (vegetables, fruit, and water) or discouraged foods and fluids (foods hight in salt, sugar, and fat, and carbonated sweet drinks). There was a decrease in fruit and vegetable consumption in terms of daily and weekly basis, while the consumption of water declined from baseline to follow-up. Conversely, discouraged food (non-core) consumption, already high at baseline, further increased during the lockdown.

### Physiological measures

Inspection of the data from baseline to follow-up for the physiological measures with Pearson’s product moment test-retest (TR) correlations indicated moderate to strong relationships: (i) HbA1C = 0.85, *p* <. 001; (ii) BMI percentiles = 0.79, *p* <. 001; (iii) Height = 0.99, *p* <. 001; and Weight = 0.98, *p* <. 001 (strong = 0.80 or above and moderate = 0.50 to 0.79; [48; 49]. There was a small increase in child height from baseline to follow-up (*t* = -4.79, *p* < .001, *d* = 0.06) but no changes in their weight. Consequently, there was also a small decrease in their BMI percentiles (*t* = 2.20, *p* = .031, *d* = 0.17). It is worth noting that the anthropometric measures at follow-up may not have been fully up to date, because outpatient visits were curtailed by the pandemic.

### Reliable change indices for well-being and mental health

The findings for the repeated measures *t*-tests showed that the mental health and well-being of parents and children worsened during the pandemic lockdown. No notable changes were found for the lifestyle related variables so they were not explored in the regression analysis.

### Children’s well-being at follow-up

A hierarchical regression was run to predict children’s well-being scores at follow-up after inspecting the pattern of correlates for the RCIs and those for the follow-up scores (see Table 5). At step one, children’s baseline well-being scores were controlled for and accounted for 10% of the unique variance in children’s follow-up well-being scores (*F* (1, 68) = 7.54, *p* = .008). The addition of the five predictor variables in step two accounted for an additional 27% of the unique variance (*F* (5, 63) = 5.41, *p* < .001) and the full model accounted for 37% of the unique variance in children’s well-being follow-up scores (*F* (6, 63) = 6.17, *p* < .001, post hoc power = 0.99 and Cohen’s *f*-squared = 0.59). Examination of the VIF (1.00 to 1.96) and tolerance (0.51 to 1.00) values indicated that there were no violations of regression diagnostic assumptions.

**Table 5:** *Regression coefficients for Predicting Children’s Well-being at Follow-Up; parental outcomes; and Parental Mental Health RCI scores*

Outcome variable	Predictor variables	β	*p*
**Children’s Well-being at Follow-Up**			
Well-being at FU			
Step 1	Well-being at BL	316	0.008
Step 2	Difficulties RCI scores	− 0.291	0.042
	HFS worry RCI scores	− 0.131	0.217
	Separation anxiety RCI scores	0.203	0.059
	Depressive problem RCI scores	− 0.004	0.976
	Coping 3 RCI scores	− 0.284	0.007
*Parental Outcomes*			
Shame RCI scores	Parenting style sum RCI scores	0.186	0.105
	DASS-21 stress RCI scores	− 0.201	0.067
	Child feeding sum RCI scores	0.334	0.005
Parenting Style Sum RCI scores			
	Shame RCI scores	0.188	0.137
	HFS behaviour RCI scores	0.153	0.188
	Child feeding sum RCI scores	0.240	0.059
Child Feeding Sum RCI scores	Shame RCI scores	0.346	0.003
	DASS-21 depression RCI scores	− 0.182	0.089
	Parenting style sum RCI scores	0.225	0.047
*Parental Mental Health RCI Scores*			
DASS-21 Depression RCI scores			
	Child feeding sum RCI scores	− 0.240	0.045
DASS-21 Anxiety RCI scores			
	HFS behaviour RCI scores	− 0.245	0.041
DASS-21 Stress RCI scores			
	Shame RCI scores	− 0.219	0.059
	HFS behaviour RCI scores	-303	0.010

### Parental outcomes at follow-up

Preliminary correlational analysis identified that the best predictors of parental outcomes were the RCIs derived from their self-report measures (e.g., DASS-21 RCI depression scores) rather than the baseline or follow-up scores, so the former were used to build three regression models of parental outcomes (see Table 5).

The model for predicting: (1) parental Shame RCI scores accounted for 26.5% of the unique variance (*F* (3, 66) = 7.93, *p* < .001, post hoc power = 0.99, Cohen’s *f*-squared = 0.36); (2) parenting style sum RCI scores accounted for 18.5% of the unique variance (*F* (3, 66) = 4.99, *p* < .01, post hoc power = 0.92, Cohen’s *f*-squared = 0.23); and (3) child feeding sum RCI scores accounted for 27.6% of the unique variance (*F* (3, 66) = 8.39, *p* < .001, post hoc power = 0.99, Cohen’s *f*-squared = 0.38). No violations of regression diagnostic assumptions occurred during the modelling of parental outcomes: VIF = 1.04 to 1.15 and Tolerance = 0.87 to 0.96 (Model 1); VIF = 1.07 to 1.26 and Tolerance = 0.79 to 0.93 (Model 2), and VIF = 1.01 to 1.13 and Tolerance 0.89 to 0.98 (Model 3).

### Parental mental health at follow up

Inspection of the parental mental health RCIs identified that three models could be derived from the data (see Table 5). In Model 1, child feeding sum RCI scores accounted for 5.8% of the unique variance in DASS-21 depression RCI scores, (*F* (1, 68) = 4.17, *p* < .05, post hoc power = 0.56, Cohen’s *f*-squared = 0.06.). In Model 2, HFS behaviour RCI scores accounted for 6.0% of the unique variance in DASS-21 anxiety RCI scores, (*F* (1, 68) = 4.36, *p* < .05, post hoc power = 0.56, Cohen’s *f*-squared = 0.06). In Model 3, shame RCI and HFS behaviour RCI scores accounted for 16.9% of the unique variance in DASS-21 stress RCI scores, (*F* (2, 67) = 6.82, *p* < .01, post hoc power = 0.92, Cohen’s *f*-squared = 0.20, VIF = 1.05, Tolerance = 0.95). Model 1 and Model 2 were found to have low post hoc power and Cohen’s *f*-squared values because of the small amount of unique variance accounted for in each model in relation to the sample size (*N* = 70). These findings would need to be replicated to reliably identify the strength and direction of the relations between the predictor and outcome variables.

### Parents’ responses to a questionnaire about the impact of COVID-19 lockdown

Table 2 summarises the distribution of sample responses from parents who answered the questionnaire about the impact of COVID-19 in 2020. It can be seen that lockdown has had a negative impact on parents’ lives in all aspects, except family finances (reflected in question number 2).

To provide more detail to the responses, each question was followed up with a prompt asking parents if they had anything to add.

**Q1**. A total of 32 parents commented. Of these, 28 stated that they missed seeing their friends and attending family gatherings; two of the parent responders were divorced and one reported that his child missed her mother. One mother reported that her husband was not in Kuwait and that she was missing a family gathering as a result; another mother divulged that her only child hates her father because of his work as a policeman which keeps him away from the home.

**Q2**. Of seven respondents, five answered that they had no present income and two reported that they had lost their employment.

**Q3**. Of the 22 parents who provided comments, five answered that they had been careless; three reported experiencing feelings of fear, whilst two others described shouting and feeling nervous regularly. Stronger anger issues were acknowledged by three of the parents; three others reported high levels of caring, concern, and associated strictness, three mothers said that they had more responsibilities in the absence of their husbands. One father said that COVID-19 had impacted negatively on the relationship between him and his child, as “he was not there when he was supposed to be.” Another mother said her behaviour had bounced between being strict and careless. “Money shortages made me feel that I could not keep up with my family expenses,” she explained.

**Q4**. A total of 29 parents commented on this question. Ten revealed they had experienced depression, weight gain, and anger issues; six reported suffering from sleep problems, stress, and nervousness. Ten had experienced anger issues, anxiousness, stress, and fear. Three of the parents said they were constantly fearful, and that life was not enjoyable anymore.

**Q5**. Twenty-eight parents commented on this question; 24 reported their children had suffered from high blood sugar; two children with high blood sugar had been admitted to hospital, and two reported levels of low blood sugar in their children.

**Q6**. Out of nine respondents, four reported being fearful of their children having high blood sugar; three revealed that their children had refused their medicine, one child was said to care more about medicine than previously, and one child insisted that they did not care about their medicine.

**Q7**. Of 34 parents who commented on the question, 10 reported that their children had experienced stress, nervousness, and a reluctance to go out in public. Five reported fearfulness and hyperactivity in their children related to COVID-19 and noted that most had many questions about it. Two reported that their children had lost interest in food and play and became careless as a consequence of emotional anxiety about the pandemic. Two reported child bedwetting and anger as symptoms of their children’s reactions to COVID-19, while another two said their children were worried about the future. Additionally, three reported their children had experienced sleep problems, nightmares, and/or that they wanted to sleep near their mothers. Two reported that their children have tended to cry a lot since the outbreak of the pandemic, have a fear of death, and are more anxious or overly sensitive. Four revealed that their children wanted to self-isolate and did not want to play outside. Three reported that their children had experienced sadness and loneliness due to the pandemic, and had missed their friends.

**Q8**. Twelve respondents commented on the question. Five reported a lack of access to hospital appointments; four reported delays in receiving medicine, two reported sleep issues and concerns about clinic closures. Lastly, one parent reported that they or their child had experienced eye problems and been refused routine care.

**Q9.** Of 29 parents who commented, most reported that their children had experienced sleep issues (including insomnia), eating issues, and a decrease in physical activities. Additionally, two reported that their children had been repeatedly asking for non-healthy food. Three parents reported that their children had experienced insomnia.

**Q10.** A total of 32 parents commented on this question. Most reported sleep issues, lack of exercise, and eating-related issues (eating significantly less or more food or refusing it). Three parents reported their children asked for non-healthy food.

**Q11.** Fifteen parents commented, of which 12 reported experiencing feelings of fear about life and the future, one reported fear of death, and two revealed they did not trust the future and worried about it.

**Q12.** Twelve parents offered comments, of which four said that life had changed for the worse, three described the challenging effects of no school, no work, and a lack of social gatherings. Two parents said they no longer experienced enjoyment in their lives and another two reported major changes in their thoughts and beliefs about life. Only one parent reported losing their employment, and as a result, a major impact on their daily routine.

## Discussion

Our findings clearly show that the Covid-19 pandemic lockdown had a significant impact on the mental health, well-being, and lifestyle of 9–12 year old children with Type 1 diabetes, and their parents. Children had higher scores than before the lockdown with respect to mental health issues, such as anxiety, depression, low self-esteem, and stress. These issues were probably related to daily routine disruption and impairment of quality of life. Although we were unable to recruit a control cohort, the existing research indicates that children with Type 1 diabetes may be more vulnerable than their healthy peers to developing fear, distraction, and irritability [15]. Imran et al. [50] suggested that children in quarantine are likely to have anxiety and stress due to social isolation, fear of an unknown disease, and stigmatisation. Younger children may be more vulnerable to adverse circumstances, and in our previous research we have shown that primary-age children with Type 1 diabetes showed poorer mental health and lower wellbing than their healthy counterparts, prior to the pandemic [9], which could have made them more susseptible to stress and challenges of the lockdowns.

We also recorded higher levels of HbA1c during the lockdown - the adverse change in diabetes status was seen in almost a third of our sample, who transited from managed to unmanaged classification. Passanisi et al. [15] also reported higher levels of blood sugar during the lockdown. Due to limited access to health services, patients were unable to keep scheduled outpatient follow-up appointments and were also forced to change their approach to chronic disease management.

Our study observed lower scores for well-being in both parents and children. The regression analyses showed that child well-being at follow-up could be predicted by changes in SDQ difficulities RCI scores, *T*-scored RCADS separation anxiety RCI scores, raw CBCL depressive problem RCI scores, and two parental variables, namely; HFS worry RCI scores, and coping 3 RCI scores, after controlling for baseline well-being scores. A similar study by McArthur et al. [51] found that Covid-19 has the potential for significant negative consequences on children’s mental health and well-being.

Alongside sleep disorders, adverse changes in lifestyle that included fewer physical activities and minimal consumption of healthy core food were also observed from baseline to follow-up. Such negative health effects may be exacerbated if children are confined to their homes without access to outdoor activities or interactions with friends during the outbreak. An expected decrease in exercise and increase in sedentary behaviour could thus have a negative impact on glycemic control [52]. A previous study suggested that there is a correlation between physical health outcomes and well-being in improving immune system response, and in several ways, the lockdown was pushing children with Type 1 diabetes and their parents out of balance regarding their well-being [53]. The sudden change in lifestyle along with distance learning has led people to play more video games, modify their eating habits, and sleep time; this could contribute to an increase in weight gain, and to excessive consumption of snacks and unhealthy food. Di Renzo [54] suggested that eating due to stress or boredom is one of the many implications of the Covid-19 lockdown. In our previous research, we have found a significant relationship between BMI and mental health, including low academic self-esteem, depression, and anxiety, in the diabetes cohort prior to the pandemic [9]. In the present study, we recorded a slight decrease in the BMI incogruent with the poorer eating habits and increase in sedentary behaviour reported at follow-up; this was probably due to lack of tracking of antropomethric variables in the outpatient clinics, during their reduced service.

Our present study found that parents’ worry increased while adaptive health coping decreased. A similar finding by Sweenie, Mackey and Streisand [55] suggested that increasing parental stress could be associated with their child’s diabetes condition, such as fear of hypoglycaemia. Moreover, Al-Abdulrazzaq et al. [21] found that children with Type 1 diabetes in Kuwait experienced a decline in health-related quality of life following the pandemic lockdown, and their parents had negative caring experiences. Another Kuwaiti study found that in comparison to men, women are more likely to experience a decline in mental health during the pandemic with depression being statistically significantly higher between females and males [19]. By contrast, differences were observed between previous studies, and the current study, as parents’ external shame decreased during the lockdown. The regression analysis for predicting parental outcomes suggests that there is a relation between changes in shame RCI scores, parenting style RCI scores, stress RCI scores, and child feeding RCI scores. External factors are theorized to be the source of shame, due to the unique nature of lockdown and self-isolation, there is a probability that external shame was reduced to its minimum level in this study [56; 57]. Moreover, shame is characterised by a negative self-evaluation and is linked to avoidance behaviours or avoidance-oriented behavioural intention [57].

Parents of children with Type 1 diabetes also showed increased levels of depression, anxiety, and stress at follow-up compared to baseline, with average scores falling into a clincial range. This finding is also supported by the regression analyses that show DASS-21 depression RCI scores, DASS-21 anxiety RCI scores, and DASS-21 stress RCI scores are predicted by changes in child feeding sum RCI scores, HFS behaviour RCI scores, and shame RCI scores. Our finding is in line with the existing literature as it has been suggested that social restrictions, working from home, homeschooling, and changes to everyday family life increased parenting stress, anxiety, and depression [58].

Overall, our findings stronly suggest that there is a need for a psychological intervention for children and their parents to overcome the implications caused by the lockdown and restore an appropriate balance regarding their physical, psychological, and mental health. This would require attention from multi-disciplinary specialised teams (e.g., medical professionals, researchers, psychiatrists, psychologists, and psychotherapists) to identify those in need of help, and to offer appropriate treatment and monitoring.

This study has some limitations. We were unable to collect face-to-face data at follow-up. It could be that parents and children were more (or less) frank about their experiences when interviewed by phone than would have been the case if the interviews were conducted face-to-face, like they were at baseline. Second, because of lack of long-term research with comparable samples, especially at younger primary age, we do not know whether children’s and parents’ scores would have been expected to change over a year, in the absence of a pandemic, and in which direction. However, we consider that such dramatic changes such as we have reported in the present paper could not come about simply because of these reasons.

A major strength of the present study is being able to offer a prospective, rather than retrospective, research into the effects of the pandemic lockdown. To our knowledge, there have been no comparable reports in the published literature. Comparing the scores on a diverse range of psychological variables, and reporting how they may be related, is another strength of this study. Finally, our participants were younger than those typically included in Type 1 diabetes studies – an underserved population which ought to be included in research and provision, given that mental health problems often start in early and middle childhood.

In conclusion, we have found that the COVID-19 lockdown experience had a significant adverse impact on the mental health, well-being, and lifestyle of the 9–12 year old children with Type 1 diabetes and their parents in Kuwait. Following the first lockdown, there were further restrictions and limitation imposed on the same population, similarly to other countries. Although lockdown restrictions have stopped in the past two years, post-pandemic stressors may be expected to continue to adversely affect this cohort. Further research on these long-term outcomes is needed.

## Electronic supplementary material

Below is the link to the electronic supplementary material.

Supplementary Material 1

## Data Availability

Full data set is available from 10.5281/zenodo.7014722. This paper has not been submitted for publication elsewhere. An earlier version of it has been presented as part of PhD thesis: https://research.bangor.ac.uk/portal/files/52728852/2023_Alazmi_A_PhD_PDF.pdf.

## Abbreviations

WHO: World Health Organization
BMI: Body Mass Index
HbA1c (Glycated hemoglobin): Is the standard medical measure of average blood sugar concentration over the period of 8–12 weeks
ISPAD: International Society for Pediatric and Adolescent Diabetes
CSEI: Coppersmith Self-Esteem Inventory-School Form
KEDS: Kids Eating Disorder Survey
WHO-5: WHO-5 Well-being Index Questionnaire
RCADS: Revised Child Anxiety and Depression Scale
CODI: Coping Questionnaire for Children and Adolescents
CBCL: Child Behavior Checklist
SDQ: Strengths and Difficulties Questionnaire
CSHQ-A: Child’s Sleep Habits Questionnaire
LBCL: Lifestyle Behaviour Checklist
CDQ: Children’s Dietary Questionnaire
C-PAQ: Physical Activity Questionnaire for Children
OaS: Other as a Shamer scale
CHIP: Coping Health Inventory for Parents
HFS-P: Hypoglycaemia Fear Survey
DASS-21: Depression Anxiety Stress Scales
CFQ: Child Feeding Questionnaire
SPSS: Statistical Package for the Social Sciences version 25
RCIs: Reliable Change Indices
TR: Pearson’s product moment test-retest
