# Supplementary material for: Changes in health, lifestyle, and wellbeing of children with type 1 diabetes and their parents during the pandemic
Source: BMC Psychol. 2024 Oct 26;12:593. doi: 10.1186/s40359-024-02102-z (PMC11514897; doi:10.1186/s40359-024-02102-z)
Supplement: Supplementary file 1 — Supplementary Material 1 [file 40359_2024_2102_MOESM1_ESM.docx]

**Supplement**

**Descriptive Statistics Before Covid 19 (baseline)**

Table S1

*Descriptive Statistics for* *Child Self-Completed Measures*

| *Measure* | *N* | Min | Max | *M* | *SD* | Skew | Kurtosis |
| --- | --- | --- | --- | --- | --- | --- | --- |
| Child Weight in KG | 70 | 25.00 | 106.00 | 40.06 | 15.18 | 2.67 | 8.68 |
| Child Height in CM | 70 | 125.00 | 163.00 | 140.00 | 9.49 | 0.62 | -0.29 |
| BMI Percentiles | 70 | 14.00 | 99.00 | 74.95 | 19.73 | -0.85 | 0.43 |
| Child Well-Being | 70 | 9.00 | 25.00 | 17.45 | 4.53 | -0.12 | -0.99 |
| *KEDS* |  |  |  |  |  |  |  |
| Eating Disorders | 70 | 0.00 | 8.00 | 4.64 | 2.56 | -0.56 | -1.07 |
| Binge Eating | 70 | 0.00 | 2.00 | 0.40 | 0.66 | 1.42 | 0.74 |
| Body Dissatisfaction | 70 | -1.00 | 4.00 | 1.28 | 1.27 | 0.26 | -0.78 |
| *Coping* |  |  |  |  |  |  |  |
| Acceptance | 70 | 6.00 | 30.00 | 18.42 | 6.35 | 0.04 | -0.60 |
| Avoidance | 70 | 4.00 | 20.00 | 11.72 | 3.50 | 0.21 | -0.09 |
| Cognitive-Palliative | 70 | 9.00 | 25.00 | 17.85 | 3.12 | -0.34 | 1.10 |
| Distance | 70 | 4.00 | 18.00 | 10.22 | 3.64 | -0.22 | -0.84 |
| Emotional Reaction | 70 | 7.00 | 30.00 | 20.81 | 4.34 | -0.61 | 0.72 |
| Wishful Thinking | 70 | 3.00 | 15.00 | 10.88 | 2.77 | -0.26 | -0.16 |
| *Coppersmith Self-Esteem* |  |  |  |  |  |  |  |
| General Self | 70 | 6.00 | 25.00 | 12.20 | 3.25 | 0.77 | 2.376 |
| Social Self | 70 | 0.00 | 7.00 | 3.41 | 1.70 | 0.24 | -0.67 |
| Home Parents | 70 | 0.00 | 7.00 | 3.25 | 1.51 | 0.56 | 0.22 |
| School Academic | 70 | 0.00 | 7.00 | 3.18 | 1.56 | 0.33 | -0.33 |
| Total Score | 70 | 6.50 | 22.00 | 11.02 | 2.85 | 0.80 | 1.64 |
| *RCADS* |  |  |  |  |  |  |  |
| Social Phobia | 70 | 27.00 | 64.00 | 42.91 | 7.73 | 0.25 | 0.53 |
| Panic Disorder | 70 | 36.00 | 82.00 | 55.70 | 9.21 | 0.08 | 0.15 |
| Major Depression | 70 | 32.00 | 83.00 | 51.80 | 9.30 | 0.79 | 1.26 |
| Separation Anxiety | 70 | 36.00 | 77.00 | 55.73 | 8.27 | 0.07 | -0.11 |
| Generalized Anxiety | 70 | 29.00 | 64.00 | 44.23 | 7.34 | 0.33 | 0.46 |
| Obsessive Compulsive | 70 | 5.00 | 72.00 | 48.43 | 10.46 | -0.82 | 3.59 |

Table S2

*Descriptive Statistics for* *Parent-Completed Self-Report Measures*

| Measure | *N* | Min | Max | *M* | *SD* | Skew | Kurtosis |
| --- | --- | --- | --- | --- | --- | --- | --- |
| *Parenting Scale* |  |  |  |  |  |  |  |
| Laxness | 70 | 1.91 | 6.55 | 4.32 | 0.82 | -0.23 | 1.39 |
| Over Reactivity | 70 | 1.60 | 6.30 | 4.27 | 0.86 | -0.38 | 0.73 |
| Verbosity | 70 | 1.00 | 6.57 | 4.14 | 1.13 | -0.32 | -0.09 |
| Parenting Scale Sum | 70 | 2.03 | 6.30 | 4.31 | 0.70 | -0.63 | 2.68 |
| *Child Feeding Questionnaire* |  |  |  |  |  |  |  |
| Responsibility | 70 | 1.00 | 5.00 | 4.00 | 0.95 | -1.40 | 2.28 |
| Parental Weight | 70 | 1.50 | 4.00 | 3.08 | 0.55 | -0.53 | 0.17 |
| Child Weight | 70 | 2.00 | 4.00 | 2.92 | 0.55 | 0.01 | -0.72 |
| Concern About Child Weight | 70 | 1.00 | 5.00 | 3.39 | 0.94 | -0.14 | -0.66 |
| Restriction | 70 | 2.13 | 5.00 | 3.75 | 0.73 | -0.35 | -0.36 |
| Pressure to Eat | 70 | 1.00 | 5.00 | 3.17 | 0.96 | -0.17 | -1.07 |
| Monitoring | 70 | 2.00 | 5.00 | 3.70 | 0.84 | -0.36 | -0.53 |
| Shame Total | 70 | 31.00 | 72.00 | 56.21 | 10.05 | -0.33 | -0.63 |
| *DASS-21* |  |  |  |  |  |  |  |
| Stress | 70 | 0.00 | 17.00 | 7.42 | 3.78 | 0.59 | 0.04 |
| Anxiety | 70 | 0.00 | 16.00 | 6.42 | 3.91 | 0.41 | -0.25 |
| Depression | 70 | 0.00 | 14.00 | 5.20 | 3.30 | 0.43 | -0.05 |
| *Parent Well-Being* | 70 | 2.00 | 25.00 | 15.64 | 5.62 | -0.39 | -0.07 |
| *Coping* |  |  |  |  |  |  |  |
| Subscale 1 | 70 | 26.00 | 55.00 | 42.32 | 7.04 | -0.23 | -0.69 |
| Subscale 2 | 70 | 23.00 | 51.00 | 39.30 | 6.80 | -0.70 | -0.08 |
| Subscale 3 | 70 | 12.00 | 24.00 | 17.52 | 2.80 | 0.17 | -0.43 |

Table S3

*Descriptive Statistics for* *Parent-Completed Child Measures*

| *Measure* | *N* | Min | Max | *M* | *SD* | Skew | Kurtosis |
| --- | --- | --- | --- | --- | --- | --- | --- |
| *Sleep Habits* |  |  |  |  |  |  |  |
| Sleep Bedtime | 70 | 6.00 | 25.00 | 14.82 | 4.32 | 0.28 | -0.25 |
| Sleep Behaviour | 70 | 4.00 | 22.00 | 9.37 | 3.59 | 1.06 | 1.43 |
| Waking During the Night | 70 | 0.00 | 8.00 | 3.75 | 1.40 | -0.00 | 1.47 |
| Morning Wake Up | 70 | 0.00 | 16.00 | 7.70 | 3.08 | 0.21 | 0.24 |
| *Fear of Hypoglycaemia* |  |  |  |  |  |  |  |
| Behaviour Scale | 70 | 1.10 | 5.00 | 3.82 | 0.80 | -1.19 | 1.81 |
| Worry Scale | 70 | 1.06 | 4.25 | 2.89 | 0.69 | -0.49 | -0.01 |
| *Life style Behaviour Checklist* |  |  |  |  |  |  |  |
| Food | 70 | 4.00 | 45.00 | 21.55 | 8.91 | 0.61 | 0.29 |
| Physical Activity and Situation | 70 | 1.00 | 45.00 | 12.68 | 7.39 | 1.29 | 4.13 |
| *CBCL* |  |  |  |  |  |  |  |
| Depressive Problem | 70 | 0.00 | 15.00 | 5.45 | 3.03 | 0.67 | 0.49 |
| Anxiety Problems | 70 | 0.00 | 10.00 | 4.45 | 2.06 | 0.31 | -0.02 |
| Anxious ‎/ Depressed | 70 | 0.00 | 12.00 | 6.12 | 2.75 | 0.29 | -0.15 |
| Withdrawn ‎/ Depressed | 70 | 0.00 | 10.00 | 3.60 | 2.62 | 0.67 | -0.13 |
| *SDQ* |  |  |  |  |  |  |  |
| Emotional Symptoms | 70 | 0.00 | 8.00 | 2.91 | 1.80 | 0.63 | 0.56 |
| Conduct Problem | 70 | 1.00 | 6.00 | 2.77 | 1.11 | 0.15 | -0.31 |
| Hyperactivity | 70 | 0.00 | 5.00 | 2.28 | 1.38 | 0.34 | -0.64 |
| Peer problem | 70 | 0.00 | 7.00 | 3.70 | 1.26 | -0.33 | 0.63 |
| Difficulties Global Score | 70 | 2.00 | 20.00 | 11.67 | 3.59 | 0.21 | 0.16 |
| Prosocial | 70 | 0.00 | 9.00 | 3.70 | 2.20 | 0.49 | -0.22 |
| *Children’s Dietary Questionnaire* |  |  |  |  |  |  |  |
| Fruits Eaten in the Last 7 Days | 70 | 0.00 | 16.00 | 6.05 | 3.45 | 0.37 | 0.07 |
| Fruit Last 24 Hours | 70 | 0.00 | 10.00 | 2.01 | 1.69 | 1.80 | 6.12 |
| Fruit Last Week | 70 | 0.00 | 6.00 | 2.81 | 1.47 | -0.14 | -0.93 |
| Vegetable Eaten in the Last 7 Days | 70 | 0.00 | 14.00 | 5.17 | 3.10 | 0.54 | 0.57 |
| Vegetable in Evening Meal in the Last 24 Hours | 70 | 0.00 | 5.00 | 0.98 | 0.98 | 1.71 | 4.13 |

Table 3 continued

| Measure | *N* | Min | Max | *M* | *SD* | Skew | Kurtosis |
| --- | --- | --- | --- | --- | --- | --- | --- |
| Vegetable Last 24 Hours | 70 | 0.00 | 5.00 | 0.85 | 0.93 | 1.81 | 5.55 |
| Vegetable Last Week | 70 | 0.00 | 6.00 | 2.25 | 1.48 | 0.61 | -0.55 |
| Diary - Last 24 Hours | 70 | 0.00 | 17.00 | 6.28 | 3.62 | 1.06 | 1.30 |
| Diary Reduced -Last 24 Hours | 70 | 0.00 | 7.00 | 1.85 | 1.58 | 1.14 | 1.33 |
| Non-Core Foods Last 7 Days | 70 | 6.00 | 53.00 | 22.38 | 9.09 | 1.21 | 2.19 |
| Sweetened Beverage Last 24 Hours | 70 | 0.00 | 5.00 | 1.01 | 1.09 | 1.39 | 2.28 |
| Water Last 24 Hours | 70 | 1.00 | 5.00 | 4.28 | 0.83 | -1.96 | 5.70 |
| Fruit Eaten Average Daily Portion | 70 | 0.00 | 3.00 | 1.55 | 0.67 | -0.17 | -0.36 |
| Vegetable Eaten Average Daily Portion | 70 | 0.00 | 3.57 | 1.32 | 0.63 | 0.46 | 1.41 |
| Non-Core Foods Average Daily Portion | 70 | 0.86 | 7.57 | 3.19 | 1.29 | 1.21 | 2.19 |

| *Children’s Physical Activity Questionnaire* |  |  |  |  |  |  |  |
| --- | --- | --- | --- | --- | --- | --- | --- |
| Physical Activity Frequency Weekday | 70 | 0.00 | 6.00 | 2.22 | 1.99 | 0.31 | -1.04 |
| Physical Activity Total Time in Minutes Weekdays | 70 | 0.00 | 120.00 | 39.64 | 37.31 | 0.50 | -0.77 |
| Physical Activity Weekend Frequency | 70 | 0.00 | 0.00 | 0.00 | 0.00 | . | . |
| Physical Activity Weekend Total Time in Minutes | 70 | 0.00 | 0.00 | 0.00 | 0.00 | . | . |
| Leisure Activity Weekday Frequency | 70 | 0.00 | 0.00 | 0.00 | 0.00 | . | . |
| School Active Travel | 70 | 0.00 | 0.00 | 0.00 | 0.00 | . | . |
| School Activity Total Time in Minutes | 70 | 0.00 | 45.00 | 0.64 | 5.37 | 8.36 | 70.00 |
| Sedentary behaviours Total Frequency | 70 | 0.00 | 3.00 | 1.42 | 0.64 | -0.05 | -0.19 |
| Sedentary Behaviours Total Time in Minutes Weekdays | 70 | 0.00 | 500.00 | 192.00 | 120.34 | 0.41 | -0.03 |
| Sedentary Behaviours Total Time in Minutes Weekend | 70 | 0.00 | 1000.00 | 515.8 | 211.43 | 0.25 | -0.52 |

**Descriptive Statistics After Covid 19 (follow-up)**

Table S4

*Descriptive Statistics for* *Child Self-Completed Measures*

| *Measure* | *N* | Min | Max | *M* | *SD* | Skew | Kurtosis |
| --- | --- | --- | --- | --- | --- | --- | --- |
| Child Weight in KG | 70 | 27.00 | 28.00 | 39.81 | 11.88 | 1.92 | 4.25 |
| Child Height in CM | 70 | 125.00 | 163.00 | 140.41 | 9.17 | 0.69 | -0.26 |
| BMI Percentiles | 70 | 20.00 | 99.00 | 71.44 | 20.95 | -0.71 | 0.24 |
| Child Well-Being | 70 | 9.00 | 18.00 | 12.9 | 2.22 | 0.10 | -0.67 |
| *KEDS* |  |  |  |  |  |  |  |
| Eating Disorders | 70 | 0.00 | 8.00 | 4.28 | 2.63 | -0.35 | -1.28 |
| Binge Eating | 70 | 0.00 | 2.00 | 0.41 | 0.67 | 1.36 | 0.58 |
| Body Dissatisfaction | 70 | -1.00 | 5.00 | 1.85 | 1.15 | -0.17 | 0.45 |
| *Coping* |  |  |  |  |  |  |  |
| Acceptance | 70 | 6.00 | 30.00 | 17.04 | 5.90 | 0.37 | -0.45 |
| Avoidance | 70 | 4.00 | 20.00 | 11.22 | 3.18 | 0.37 | 0.65 |
| Cognitive-Palliative | 70 | 6.00 | 25.00 | 16.90 | 3.67 | -0.52 | 0.49 |
| Distance | 70 | 4.00 | 18.00 | 10.40 | 3.63 | -0.24 | -0.67 |
| Emotional Reaction | 70 | 9.00 | 30.00 | 20.21 | 4.31 | -0.05 | -0.25 |
| Wishful Thinking | 70 | 6.00 | 20.00 | 12.98 | 3.13 | -0.06 | -0.63 |
| *Coppersmith Self-Esteem* |  |  |  |  |  |  |  |
| General Self | 70 | 1.00 | 18.00 | 10.19 | 3.31 | -0.36 | 0.45 |
| Social Self | 70 | 0.00 | 7.00 | 3.37 | 1.76 | -0.01 | -0.78 |
| Home Parents | 70 | 0.00 | 7.00 | 3.12 | 1.59 | 0.15 | -0.55 |
| School Academic | 70 | 0.00 | 6.00 | 2-97 | 1.48 | 0.13 | -0.71 |
| Total Score | 70 | 0.50 | 17.50 | 10.18 | 3.12 | -0.42 | 0.71 |
| *RCADS* |  |  |  |  |  |  |  |
| Social Phobia | 70 | 31.00 | 76.00 | 49.58 | 8.48 | 0.34 | 0.56 |
| Panic Disorder | 70 | 49.00 | 96.00 | 72.12 | 10.27 | 0.22 | -0.01 |
| Major Depression | 70 | 20.00 | 97.00 | 70.77 | 13.28 | -0.76 | 2.27 |
| Separation Anxiety | 70 | 45.00 | 106.00 | 72.22 | 12.34 | 0.58 | 0.97 |
| Generalized Anxiety | 70 | 43.00 | 77.00 | 56.27 | 8.82 | 0.98 | -0.04 |
| Obsessive Compulsive | 70 | 35.00 | 81.00 | 59.55 | 8.71 | 0.14 | 0.17 |

Table S5

*Descriptive Statistics for* *Parent-Completed Self-Report Measures*

| Measure | *N* | Min | Max | *M* | *SD* | Skew | Kurtosis |
| --- | --- | --- | --- | --- | --- | --- | --- |
| *Parenting Scale* |  |  |  |  |  |  |  |
| Laxness | 70 | 1.91 | 5.18 | 3.90 | 0.67 | -0.56 | 0.12 |
| Over Reactivity | 70 | 1.60 | 5.30 | 3.88 | 0.67 | -0.44 | 0.78 |
| Verbosity | 70 | 2.00 | 6.57 | 3.80 | 0.84 | 0.21 | -0.39 |
| Parenting Scale Sum | 70 | 1.84 | 5.25 | 3.86 | 0.59 | -0.62 | 1.03 |
| *Child Feeding Questionnaire* |  |  |  |  |  |  |  |
| Responsibility | 70 | 1.00 | 5.00 | 3.70 | 0.79 | -1.23 | 1.99 |
| Parental Weight | 70 | 2.00 | 4.25 | 3.29 | 0.51 | -0.71 | 0.63 |
| Child Weight | 70 | 1.80 | 4.00 | 2.81 | 0.50 | 0.08 | -0.51 |
| Concern About Child Weight | 70 | 1.00 | 4.67 | 3.09 | 0.80 | -0.42 | -0.20 |
| Restriction | 70 | 1.00 | 4.88 | 3.15 | 0.76 | -0.35 | -0.86 |
| Pressure to Eat | 70 | 1.00 | 5.00 | 3.02 | 0.96 | -0.29 | -0.14 |
| Monitoring | 70 | 1.00 | 5.00 | 3.25 | 0.93 | -0.28 | 0.25 |
| Shame Total | 70 | 27.00 | 62.00 | 49.77 | 6.60 | -0.72 | 0.72 |
| *DASS-21* |  |  |  |  |  |  |  |
| Stress | 70 | 4.00 | 21.00 | 12.87 | 3.38 | -0.18 | -0.12 |
| Anxiety | 70 | 1.00 | 21.00 | 12.35 | 3.12 | -0.43 | 2.25 |
| Depression | 70 | 2.00 | 21.00 | 12.02 | 3.31 | 0.04 | 0.82 |
| *Parent Well-Being* | 70 | 2.00 | 16.00 | 10.07 | 3.46 | -0.34 | -0.94 |
| *Coping* |  |  |  |  |  |  |  |
| Subscale 1 | 70 | 27.00 | 55.00 | 37.02 | 5.62 | 0.36 | 0.30 |
| Subscale 2 | 70 | 22.00 | 49.00 | 34.00 | 5.63 | 0.59 | -0.18 |
| Subscale 3 | 70 | 9.00 | 21.00 | 13.31 | 2.45 | 0.48 | 0.50 |

Table S6

*Descriptive Statistics for* *Parent-Completed Child Measures*

| *Measure* | *N* | Min | Max | *M* | *SD* | Skew | Kurtosis |
| --- | --- | --- | --- | --- | --- | --- | --- |
| *Sleep Habits* |  |  |  |  |  |  |  |
| Sleep Bedtime | 70 | 8.00 | 28.00 | 18.01 | 5.06 | -0.24 | -0.69 |
| Sleep Behaviour | 70 | 3.00 | 23.00 | 15.21 | 4.03 | -0.67 | 0.18 |
| Waking During the Night | 70 | 2.00 | 8.00 | 4.90 | 1.34 | 0.29 | 0.16 |
| Morning Wake Up | 70 | 4.00 | 16.00 | 10.82 | 3.04 | -0.19 | -0.43 |
| *Fear of Hypoglycaemia* |  |  |  |  |  |  |  |
| Behaviour Scale | 70 | 1.60 | 5.00 | 3.54 | 0.58 | -0.31 | 1.41 |
| Worry Scale | 70 | 1.50 | 4.06 | 3.19 | 0.50 | -0.67 | 1.65 |
| *Life style Behaviour Checklist* |  |  |  |  |  |  |  |
| Food | 70 | 2.00 | 44.00 | 26.00 | 7.98 | -0.30 | 1.06 |
| Physical Activity and Situation | 70 | 2.00 | 39.00 | 16.28 | 6.41 | 0.88 | 1.69 |
| *CBCL* |  |  |  |  |  |  |  |
| Depressive Problem | 70 | 2.00 | 19.00 | 11.10 | 3.77 | -0.22 | 0.00 |
| Anxiety Problems | 70 | 3.00 | 18.00 | 10.00 | 2.96 | 0.40 | 0.23 |
| Anxious ‎/ Depressed | 70 | 4.00 | 24.00 | 12.60 | 3.85 | 0.34 | 0.45 |
| Withdrawn ‎/ Depressed | 70 | 1.00 | 14.00 | 9.71 | 3.08 | -0.71 | 0.56 |
| *SDQ* |  |  |  |  |  |  |  |
| Emotional Symptoms | 70 | 1.00 | 9.00 | 4.44 | 1.69 | 0.12 | 0.56 |
| Conduct Problem | 70 | 1.00 | 9.00 | 4.97 | 1.65 | -0.20 | 0.12 |
| Hyperactivity | 70 | 0.00 | 8.00 | 4.11 | 1.74 | -0.23 | -0.27 |
| Peer Problem | 70 | 1.00 | 9.00 | 5.11 | 1.69 | -0.03 | 0.05 |
| Difficulties Global Score | 70 | 4.00 | 30.00 | 18.64 | 4.47 | -0.95 | 1.86 |
| Prosocial | 70 | 1.00 | 9.00 | 4.64 | 1.64 | 0.07 | 0.07 |
| *Children’s Dietary Questionnaire* |  |  |  |  |  |  |  |
| Fruits Eaten in the Last 7 Days | 70 | 0.00 | 1.00 | 0.27 | 0.44 | 1.05 | -0.92 |
| Fruit Last 24 Hours | 70 | 0.00 | 4.00 | 1.80 | 0.91 | 0.29 | 1.23 |
| Fruit Last Week | 70 | 0.00 | 5.00 | 1.60 | 1.33 | 0.51 | -0.31 |
| Vegetable Eaten in the Last 7 Days | 70 | 0.00 | 1.00 | 0.45 | 0.50 | 0.17 | -2.02 |
| Vegetable in Evening Meal in the Last 24 Hours | 70 | 0.00 | 3.00 | 1.01 | 0.57 | 0.93 | 3.45 |
| Vegetable Last 24 Hours | 70 | 0.00 | 3.00 | 0.85 | 0.70 | 0.71 | 0.98 |
| Vegetable Last Week | 70 | 0.00 | 5.00 | 1.27 | 0.97 | 1.63 | 3.37 |
| Diary - Last 24 Hours | 70 | 0.00 | 10.00 | 5.94 | 2.46 | -0.17 | -0.31 |
| Diary Reduced - Last 24 Hours | 70 | 0.00 | 6.00 | 1.92 | 1.24 | 0.93 | 0.71 |
| Non-core Foods Past 7 Days | 70 | 6.00 | 66.00 | 35.84 | 13.92 | 0.26 | -0.54 |
| Sweetened Beverage Last 24 Hours | 70 | 0.00 | 3.00 | 0.77 | 0.64 | 0.52 | 1.01 |
| Water Last 24 Hours | 70 | 1.00 | 5.00 | 3.72 | 1.02 | -1.11 | 1.16 |
| Fruit Eaten Average Daily Portion | 70 | 0.14 | 1.29 | 0.52 | 0.22 | 0.85 | 1.03 |
| Vegetable Eaten Average Daily Portion | 70 | 0.14 | 1.14 | 0.51 | 0.22 | 0.78 | 0.54 |
| Non-core Foods Average Daily Portion | 70 | 0.86 | 9.43 | 5.12 | 1.98 | 0.26 | -0.54 |
| *Children’s Physical Activity Questionnaire* |  |  |  |  |  |  |  |
|  |  |  |  |  |  |  |  |

Table 6 Continued

| Measure | *N* | Min | Max | *M* | *SD* | Skew | Kurtosis |
| --- | --- | --- | --- | --- | --- | --- | --- |
| Physical Activity Frequency Weekday | 70 | 0.00 | 0.00 | 0.00 | 0.00 | . | . |
| Physical Activity Total Time in Minutes Weekdays | 70 | 0.00 | 0.00 | 0.00 | 0.00 | . | . |
| Physical Activity Weekend Frequency | 70 | 0.00 | 0.00 | 0.00 | 0.00 | . | . |
| Physical Activity Weekend Total Time in Minutes | 70 | 0.00 | 0.00 | 0.00 | 0.00 | . | . |
| Leisure Activity Weekday Frequency | 70 | 0.00 | 0.00 | 0.00 | 0.00 | . | . |
| School Active Travel | 70 | 0.00 | 0.00 | 0.00 | 0.00 | . | . |
| School Activity Total Time in Minutes | 70 | 0.00 | 0.00 | 0.00 | 0.00 | . | . |
| Sedentary Behaviours Total Frequency | 70 | 0.00 | 0.00 | 0.00 | 0.00 | . | . |
| Sedentary Behaviours Total Time in Minutes Weekdays | 70 | 200 | 1000 | 521.57 | 202.50 | 0.44 | -0.76 |
| Sedentary Behaviours Total Time in Minutes Weekend | 70 | 0.00 | 750 | 302.42 | 134.77 | 0.67 | 0.98 |

Table S7

*Cronbach Alpha for all Measures Before and After Covid*

| *Measure* | *Original Alpha* | *Alpha* at baseline | *Alpha* at follow-up |
| --- | --- | --- | --- |
| *KEDS* | .73 | .57 | .63 |
| *Coping (For Children)* | .63 - .83 | .61 | .74 |
| *Coppersmith Self-Esteem* | .78 | .70 | .73 |
| *RCADS* | .76 - .95 | .86 | .90 |
| *Child Well-Being* | .89 | .86 | .56 |
| *Parenting Scale* | .82 | .68 | .67 |
| *Child Feeding Questionnaire* | .70 | .85 | .86 |
| Shame Total | .92 | .06 | .71 |
| *DASS-21* | .72 | .79 | .87 |
| *Parent Well-Being* | .89 | .77 | .86 |
| *Coping* | .79 | .84 | .85 |
| *Sleep Habits* | .78 - .93 | .32 | .88 |
| *Fear of Hypoglycaemia* | .89 | .91 | .89 |
| *Lifestyle Behaviour Checklist* | .90 | .89 | .83 |
| *CBCL* | .78 - .84 | .77 | .78 |
| *SDQ* | .70 - .84 | .50 | .69 |
| *Children’s Dietary Questionnaire* | .51 - .90 | .74 | .82 |
| *Children’s Physical Activity Questionnaire* | .40 | .40 | .87 |

Table S8

*Clinical Cut-Offs of Different Child Measures Used in the Present Study and Number of Participants (N=70) that Fell into Each Range.*

| *Measure* | Clinical cut-off | Children at baseline | Children at follow up (70) |
| --- | --- | --- | --- |
| *KEDS* | - | - | - |
| *Coping* | - | - | - |
| *Coppersmith Self-Esteem* | - | - | - |
| *RCADS* | > 65Indicate Borderline  > 70 Indicate Clinical |  |  |
| Social Phobia |  | 70 Normal | 68 Normal  2 Borderlines |
| Panic Disorder |  | 60 Normal  5 Borderlines  5 Clinical | 10 Normal  20 Borderlines  40 Clinical |
| Major Depression |  | 62 Normal  3 Borderlines  5 Clinical | 21 Normal  16 Borderlines  36 Clinical |
| Separation Anxiety |  | 60 Normal  8 Borderlines  2 Clinical | 15 Normal  17 Borderlines  38 Clinical |
| Generalized Anxiety |  | 70 Normal | 61 Normal  7 Borderlines  2 Clinical |
| Obsessive Compulsive |  | 66 Normal  2 Borderlines  2 Clinical | 60 Normal  5 Borderlines  5 Clinical |
| *Child Well-Being* | 25 Representing Best Possible Quality of Life  13Indicate Poor Well-Being | 54 Normal  3 Representing Best Possible Quality of Life  13 Indicate Poor Well-Being | 26 Normal  44 Indicate Poor Well-Being |
| *Sleep Habits* | - | - | - |
| *Fear of Hypoglycaemia* | - | - | - |
| *Lifestyle Behaviour Checklist* | - | - | - |
| *CBCL* |  |  |  |
| Depressive Problem | 6 Subclinical  8 Clinical | 43 Normal  11 Subclinical  17 Clinical | 7 Normal  6 Subclinical  57 Clinical |
| Anxiety Problems | 6 Subclinical  8 Clinical | 47 Normal  18 Subclinical  5 Clinical | 4 Normal  9 Subclinical  57 Clinical |
| Anxious ‎/ Depressed | 7 Subclinical  9 Clinical | 40 Normal  19 Subclinical  11 Clinical | 4 Normal  5 Subclinical  61 Clinical |
| Withdrawn ‎/ Depressed | 5 Subclinical  6 Clinical | 48 Normal  6 Subclinical  16 Clinical | 4 Normal  1 Subclinical  65 Clinical |
| *Children’s Dietary Questionnaire* | - | - | - |
| *SDQ* |  |  |  |
| \| Emotional Symptoms \| \| --- \| | 0-3 Normal  4 Borderline  5-10 Abnormal | 47 Normal  11 Borderline  Abnormal 12 | 19 Normal  13 Borderline  38 Abnormal |
| \|  \| \| --- \| \| Conduct Problem \| | 0-2 Normal  3 Borderline  4-10 Abnormal | 29 Normal  21 Borderline  20 Abnormal | 6 Normal  10 Borderline  58 Abnormal |
| \| Hyperactivity \| \| --- \| | 0-5 Normal  6 Borderline  7-10 Abnormal | 70 Normal | 55 Normal  12 Borderline  3 Abnormal |
| \| Peer Problem \| \| --- \| | 0-2 Normal  3 Borderline  4-10 Abnormal | 9 Normal  21 Borderline  40 Abnormal | 4 Normal  6 Borderline  60 Abnormal |
| \| Difficulties Global Score \| \| --- \| \|  \| | 0-13 Normal  14-16 Borderline  17-40 Abnormal | 49 Normal  15 Borderline  6 Abnormal | 8 Normal  15 Borderline  53 Abnormal |
| \| Prosocial \| \| --- \| | 0-4 Normal  5 Borderline  6-10 Abnormal | 48 Normal  9 Borderline  13 Abnormal | 32 Normal  18 Borderline  20 Abnormal |

Table 9

*Clinical Cut-Offs of Different Child Measures Used in the Present Study and Number of Participants (N=70) that Fell into Each Range.*

| *Measure* | Clinical | Parent at baseline | Parent at follow up |
| --- | --- | --- | --- |
| *Parent Well-Being* | 25 Representing Best Possible Quality of Life  13 Indicate Poor Well-Being | 44 Normal  3 Representing Best Possible Quality of Life  23 Indicate Poor Well-Being | 11 Normal  59 Indicate Poor Well-Being |
| *Parenting Scale* | - | - | - |
| *Child Feeding Questionnaire* | - | - | - |
| Shame Total | - | - | - |
| *DASS-21* |  |  |  |
| Depression | 0-4 Normal  5-6 Mild  7-10 Moderate  11-13 Severe  +14 Extremely Severe | 28 Normal  19 Mild  19 Moderate  3 Severe  1 Extremely Severe | 1 Normal  3 Mild  20 Moderate  26 Severe  20 Extremely Severe |
| Anxiety | 0-3 Normal  4-5 Mild  6-7 Moderate  8-9 Severe  +10 Extremely Severe | 14 Normal  19 Mild  12 Moderate  7 Severe  18 Extremely Severe | 1 Normal  1 Mild  2 Moderate  6 severe  60 Extremely Severe |
| Stress | 0-7 Normal  8-9 Mild  10-12 Moderate  13-16 Severe  +17 Extremely Severe | 39 Normal  12 Mild  12 Moderate  5 Severe  2 Extremely Severe | 4 Normal  10 Mild  16 Moderate  29 Severe  11 Extremely Severe |
| *Coping* | - | - | - |
